# Cell-Based Therapies for Stroke: Promising Solution or Dead End? Mesenchymal Stem Cells and Comorbidities in Preclinical Stroke Research

**DOI:** 10.3389/fneur.2019.00332

**Published:** 2019-04-09

**Authors:** Fernando Laso-García, Luke Diekhorst, Mari Carmen Gómez-de Frutos, Laura Otero-Ortega, Blanca Fuentes, Gerardo Ruiz-Ares, Exuperio Díez-Tejedor, María Gutiérrez-Fernández

**Affiliations:** Neuroscience and Cerebrovascular Research Laboratory, Department of Neurology and Stroke Center, La Paz University Hospital, Neuroscience Area of IdiPAZ Health Research Institute, Autonoma University of Madrid, Madrid, Spain

**Keywords:** aging, hypertension, diabetes, hyperglycemia, obesity, comorbidity, mesenchymal, stroke

## Abstract

Stroke is a major health problem worldwide. It has been estimated that 90% of the population attributable risk of stroke is due to risk factors such as aging, hypertension, hyperglycemia, diabetes mellitus and obesity, among others. However, most animal models of stroke use predominantly healthy and young animals. These models ignore the main comorbidities associated with cerebrovascular disease, which could be one explanation for the unsuccessful bench-to-bedside translation of protective and regenerative strategies by not taking the patient's situation into account. This lack of success makes it important to incorporate comorbidities into animal models of stroke in order to study the effects of the various therapeutic strategies tested. Regarding cell therapy, the administration of stem cells in the acute and chronic phases has been shown to be safe and effective in experimental animal models of stroke. This review aims to show the results of studies with promising new therapeutic strategies such as mesenchymal stem cells, which are being tested in preclinical models of stroke associated with comorbidities and in elderly animals.

## Introduction

**Figure 1:**
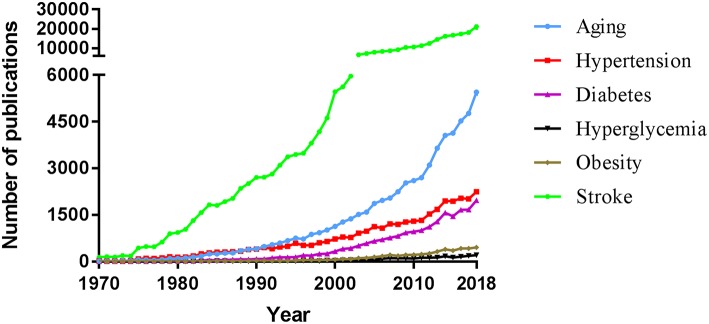
Comparative evolution of the evaluation of the effect of various comorbidities in animal models of stroke as well as in clinical research. An advanced search was performed in PubMed in December 2018 to find, for each year of publication, all articles using the text word stroke with the given term as text word: age or aging; hypertension or hypertensive or high blood pressure; diabetes or diabetes mellitus; hyperglycemia or hyperglycemic or high blood sugar or high glucose levels; or obesity or overweight. Year of final publication (and not advanced online date) of articles in English (and not other languages) was taken into account. Manual elimination of articles describing a non-comorbid stroke association were excluded.

Stroke is still the most common cause of permanent disability in adults and the second leading cause of death in the world (1). The pathology of stroke is poorly understood; however, it has been shown that the majority of patients with stroke have at least one comorbidity (2). The contribution of various risk factors to worldwide stroke burden is unknown. The INTERSTROKE study has demonstrated that five risk factors accounted for more than 80% of the global risk for all strokes (either ischemic stroke or intracerebral hemorrhage [ICH]): hypertension, current smoking, abdominal obesity, diet and physical activity (3). Furthermore, stroke incidence rises with increased in age (4). This high prevalence of comorbidities in stroke patients indicates the need for therapies in preclinical studies that take these comorbidities into account (Figure 1).

Of 502 experimental therapies for acute focal ischemic stroke, only 10% were tested in animals with hypertension. Hypertensive animals have larger infarct sizes and reduced efficacy with therapeutic intervention (5, 6). Even fewer preclinical studies assess the effects of diabetes or acute hyperglycemia on the response to therapeutic intervention (7). The majority of preclinical studies for novel therapies use young healthy animal models and this may play a role in the fact that of 1,026 treatments tested on animal models, only one has been effective in clinical trials (8).

In particular, stem cell therapy has been proven to be effective mostly in healthy animals. Various types of stem cells have been used in preclinical stroke models: embryonic stem cells, neural stem cells, induced pluripotent stem cells, mesenchymal stem cells (MSCs) and hematopoietic stem cells (9). Cell therapy has been shown to promote functional recovery, participating in processes such as immunomodulation, neurogenesis, synaptogenesis, oligodendrogenesis, axonal connectivity, and myelin formation, improvement in blood brain barrier (BBB) integrity, neovascularization and reduced lesion size, showing efficacy not only in grey matter, but also white matter injury (10–17). However, its mechanisms of action has not yet been clarified. Recent evidence has suggested that it might be related to long-distance cell-to-cell communication by paracrine function through secretory factors in the extracellular environment. Intercellular communication between stem cells and the damaged organ was thought to be regulated via the release of free molecules that transmit the signal by binding to a receptor. These molecules could in part be trophic factors, inflammation modulators and even exosomes. In order to avoid previous translation failure in stem cell therapy, STAIR guidelines suggest that further studies should be performed on animals with comorbid conditions such as hypertension and diabetes in order to improve the quality of preclinical studies of purported stroke therapies (18).

This review is focused on MSC therapies being tested in preclinical models of stroke with the most common comorbidities (hypertension, hyperglycemia, diabetes, obesity), as well as in elderly animals. We intend to provide insight into the viability of this new strategy, which could lead to an improved translation of cell therapy from bench to bedside.

## Mesenchymal Stem Cells in Preclinical Studies

### Aging in Stroke

Age is the most important risk factor for developing a stroke. Age has been proven to be a predictive factor for recovery after stroke, independent of stroke severity, characteristics and complications (19). Stroke incidence is also strongly but not solely correlated with an increase in age, in addition to the patient's general fitness (4). As previously stated, patient data shows a direct correlation between age and the occurrence of stroke. For these reasons, the impact of age should be considered carefully in preclinical studies given that the mechanisms of stroke and response to drugs can be very different in the developing, juvenile, adult and elderly brain (20). However, despite being the most important risk factor, there are currently few studies (only 7) on aged animals that evaluate the effect of administering MSC after ischemic stroke (Table 1). In this regard, systemic administration of bone marrow mononuclear cells (BMMNCs) in the acute phase after stroke reduced neurological deficits (21, 22) and reduced infarct volume, modulating post-ischemic inflammatory cytokines within the brain in older rats (21). Also in the acute phase, intravenous administration of human umbilical tissue-derived cells improved recovery of neurological function in aged rats after stroke and was associated with activation of repair processes (23). This beneficial effect of cell therapy is not only observed in the short term, but also in the long term. In one study, intra-arterial administration of bone marrow stromal cells (BMSCs) at 1 day after ischemic stroke had long-lasting beneficial effects on recovery of neurological functional (24). One interesting strategy for reducing ischemic damage to the brain would be based on a combination of therapies to act in different steps of the ischemic cascade. In this sense, the combination the granulocyte colony-stimulating factor (G-CSF) with bone marrow mesenchymal stem cells (BMMSCs) increased neurogenesis and improved microvessel recovery and density in the aged brain (25). However, another study demonstrated that the combination of G-CSF and bone marrow mononuclear cells (BMMNCs) did not further improve post-stroke recovery (26).

**Table 1:** Original studies evaluating the effect of MSC administration in ischemic stroke models using aged animals, hypertension- and diabetes-induced stroke models.

**References**	**Species**	**Stroke type**	**Cell type**	**N° Cells**	**Administration Route**	**Recovery**	**Comorbidity**
Brenneman et al. (21)	Long Evans	MCAO	BMMNC	4 × 10^6^	Intra-arterial	Improved	Aging
Coelho et al. (22)	Wistar	Focal cortical ischemia	BMMNC	3 × 10^7^	Intravenous	Improved	Aging
Zhang et al. (23)	Wistar	MCAO	HUTC	1 × 10^7^/kg	Intravenous	Improved	Aging
Shen et al. (24)	Wistar	MCAO	BMSC	2 × 10^6^	Intra-arterial	Improved	Aging
Balseanu et al. (25)	Sprague-Dawley	MCAO	G-CSF + BMMSC	50 μg/kg + 1 × 10^6^/kg	Intravenous	Improved	Aging
Buga et al. (26)	Sprague-Dawley	MCAO	G-CSF + BMMNC	50μg/kg + 1 × 10^6^/kg	Intravenous	Improved	Aging
Ito et al. (27)	SHR	Stroke prone	BMSC	5 × 10^5^	Intracranial	Not evaluated	Hypertension
Calió et al. (28)	SHR	Stroke prone	BMMSC	1 × 10^6^	Intracranial	Not evaluated	Hypertension
Kranz et al. (29)	SHR	MCAO	MSC from maternal or fetal placenta	1 × 10^6^	Intravenous	Improved	Hypertension
Minnerup et al. (30)	SHR	MCAO	BMMNC	1/5/20 × 10^6^	Intravenous	Did not improve	Hypertension
Weise et al. (31)	SHR	MCAO	HUCBMNC	8 × 10^6^/kg	Intravenous	Did not improve	Hypertension
Pösel et al. (32)	SHR	MCAO	G-CSF + BMMNC	50 μg/kg + 1.5 × 10^7^/kg	Intravenous	Did not improve	Hypertension
Taguchi et al. (33)	SHR	Focal cortical ischemia	BMMNC	5 × 10^5^	Intravenous and intraosseous	Improved	Hypertension
Wagner et al. (34)	SHR	MCAO	BMMNC	8 × 10^6^/kg	Intravenous	Did not improve	Hypertension
Wang et al. (35)	SHR	Intracerebral hemorrhage	BMMSC	1 × 10^6^	Intravenous	Improved	Hypertension
Ding et al. (36)	SHR	Intracerebral hemorrhage	BMSC	1 × 10^6^	Intracranial	Improved	Hypertension
Yan et al. (37)	Wistar	MCAO	HUCBC	5 × 10^6^	Intravenous	Improved	Diabetes Type I
Cui et al. (38)	Wistar	MCAO	BMSC	5 × 10^6^	Intravenous	Improved	Diabetes Type I
Chen et al. (39)	Wistar	MCAO	BMSC	3 × 10^6^	Intravenous	Did not improve	Diabetes Type I
Yan et al, (40)	Wistar	MCAO	BMSC + Niaspan	5 × 10^6^ + 40 mg/kg	Intravenous	Did not improve	Diabetes Type I
Ye et al. (41)	Wistar	MCAO	BMSC + Niaspan	5 × 10^6^ + 40 mg/kg	Intravenous	Not evaluated	Diabetes Type I
Yan et al. (42)	Wistar	MCAO	HUCBC	5 × 10^6^	Intravenous	Improved	Diabetes Type II
Ding et al. (43)	Wistar	MCAO	BMSC	5 × 10^7^	Intravenous	Improved	Diabetes Type II
Hu et al. (44)	Wistar	MCAO	BMSC	5 × 10^6^	Intravenous	Improved	Diabetes Type II
Xiang et al. (45)	Wistar	MCAO	BMSC-CM	10 ml/kg	Intravenous	Improved	Diabetes Type II
Yan et al. (46)	Wistar	MCAO	BMSC	5 × 10^6^	Intravenous	Improved	Diabetes Type II

*MCAO, middle cerebral artery occlusion; BMMNC, bone marrow mononuclear cell; HUTC, human umbilical tissue-derived cell; BMSC, bone marrow stromal cell; G-CSF, granulocyte colony-stimulating factor; BMMSC, bone marrow mesenchymal stem cell; SHR, spontaneously hypertensive rats; G-CSF, granulocyte colony-stimulating factor; HUCBMNC, human umbilical cord blood mononuclear cell; HUCBC, human umbilical cord blood cell; BMSC-CM, bone marrow stromal cell conditioned medium*.

Currently, all studies with MSCs in elderly animals have been performed on ischemic stroke, none on hemorrhagic stroke. Also, no standardized protocols were used in the above cell therapy studies for observing the various routes of administration and doses used. The number of studies is still very limited and further preclinical research is needed to determine the efficacy of MSCs in aged animal models. In view of the results, however, the aged brain retains the capacity for repair in response to cell therapy.

### Hypertension in Stroke

Hypertension is considered one of the most common and important vascular risk factors for stroke (3) and is responsible for approximately 52% of strokes (5); it is also closely correlated with stroke severity. Hypertension has numerous effects, such as reducing BBB integrity and promoting white matter damage and post-stroke edema (5).

In order to test a new therapeutic strategy, a good experimental animal model must first be selected. Several models have been used to induce hypertension in animals. In the past, dogs were used in experiments as a hypertension model. Currently, the rat has become the common model for research as a cost-effective alternative. There are various ways to induce hypertension in rats; for example, spontaneously hypertensive rats (SHRs), stroke-prone SHRs (SHR-SPs), endocrine hypertension by deoxycorticosterone acetate administration, angiotensin II administration and hypertension induced by stress (47–49). Based on consistent reproducibility, the SHR is probably the best model with which to observe hypertension.

Regarding cell therapy, MSCs have been used in hypertensive rats to evaluate the efficacy of functional recovery in animals (Table 1).

In cerebral ischemia, intracerebral transplantation of BMMSCs has been shown to decrease apoptotic neurons in the neocortex and ameliorate brain damage by decreasing cell death (27). This has been shown to play a primary role in brain protection. BMMSCs also act on vasculogenesis in SHR rats. Thus, the cells significantly increased the number of microvessels and their reactivity to collagen IV in the neocortex, which indicates protection of the neurovascular unit and improvement of vascular integrity (27). In this regard, BMMSCs also increase of levels of the antiapoptotic B-cell lymphoma 2 (Bcl-2) gene and decrease superoxide, demonstrating that MSC has antioxidant potential and a protective effect in SHR rats with stroke (28). Also, intravenously administered dual transplantation of human maternal or fetal placenta MSCs produces increased density of glial fibrillary acidic protein-positive cells in the area adjacent to the infarct border which may increase survival rates of regenerative astrocytes, leading to a decrease in infarct volume on day 60, triggering functional improvement in SHRs (29). However, not all studies have reported favorable functional outcomes after ischemic stroke with hypertension SHRs. BMMNCs or cryopreserved human umbilical cord blood mononuclear cells given intravenously did not show a beneficial effect on infarct volume, behavioral outcomes or inflammatory response (30, 31). Pösel et al. performed a study to determine a possible synergistic effect of G-CSF and BMMNCs after stroke in SHR rats, in which they found administration of G-CSF improved long-term functional recovery. However, this effect was negated by cotransplantation of BMMNCs as provoked splenic accumulation of granulocytes and transplanted cells, accompanied by a significant rise in circulating granulocytes and infiltration in the ischemic brain, which was detrimental to stroke outcome (32).

In addition, the Framingham Study clearly demonstrated the relevance of age and high blood pressure for lifetime risk of stroke (50), indicating the need to mimic these risk factors in preclinical stroke studies. Along these lines, animals transplanted with intravenous bone marrow cells from young SHR-SPs displayed an increase in microvasculature density in the peri-infarction zone which led to reduced ischemic brain damage and improved neurological function (33). However, in the same study BM cells led to a significant increase in levels of cytokines such as interleukin 1β (IL-1β) and monocyte chemoattractant protein 1(MCP-1) in the brain and a decrease of IL-6 levels in serum. These results suggest that modulation in the expression of inflammatory cytokines (i.e., favoring recovery/decreased inflammatory profile) did not occur and, therefore, is not likely to explain the beneficial effect of the response to cerebral ischemia observed in older SHR-SPs transplanted with BM cells from young SHR-SPs (33). Additionally, further studies reported negative results, such as the study by Wagner et al. which evaluated the therapeutic efficacy of intravenously transplanted young and aged BMMNCs in aged hypertensive rats. The authors concluded that BMMNCs from both juvenile and elderly donors failed to decrease lesion volume and functional recovery was not improved (34).

There are currently two studies in SHRs with ICH. In both studies, intravenously (35) or intracerebrally (36) transplanted BMMSCs improved neurological function and integrity of the BBB by preventing extravasation of blood through the endothelium (35, 36), resulting in improvements such as reduced brain edema and decreased cell apoptosis (36).

Although several different doses and administration routes have been used for treatment in hypertensive animals post-stroke, contradictory results have been found between different research groups using MSCs in hypertensive animals (Table 1). More studies should be performed to evaluate whether MSC therapy is effective not only in brain protection, but also in brain repair in the treatment of stroke in hypertensive animals.

### Diabetes in Stroke

Diabetes is divided into two types: type 1, in which the beta-cells of the pancreas are damaged and affected people need external administration of insulin; and type 2, which is peripheral insulin resistance, and is present in 85% of the patients with diabetes (51, 52). Diabetes causes several metabolic and pathological changes that lead to stroke including arterial stiffness, systematic inflammation, endothelial dysfunction and heart failure (53). In addition, stroke in diabetes patients increases hospital mortality (54).

There are many methods to try to mimic type 1 and 2 diabetes in rats (55). One of the most commonly used models for diabetes type 1 are the Biobreeding rats; these rats develop diabetes spontaneously or it is induced by a virus (55). Recently, however, injection of chemicals to destroy beta-cells in the islets of Langerhans has been growing in importance. Alloxan has been used for some time as a good diabetic model but it has problems such as spontaneous recovery from the diabetic condition or renal toxicity. To solve this, another beta-cytotoxic agent, streptozotocin, is used. One advantage of this chemical is that the damage is dose-dependent, which allows researchers to control the severity of that animals' hyperglycemia (56).

To replicate type 2 diabetes, various spontaneous models are used in laboratories such as the spontaneously diabetic tori rat (57), due to gradual beta-cell degeneration, or Goto-Kakizaki rats, which develop peripheral insulin resistance after 56 days (58). Another option is induction by a high fat diet and intraperitoneal streptozotocin administration, in which the rats develop hyperinsulinemia, obesity and a reduction in beta-cells (59). In addition, administration of nicotinamide intraperitoneally prior to administration of a low dose of streptozotocin protects the cells by attenuating the effect of streptozotocin (60).

Regarding MSC therapy, human umbilical cord blood cells (HUCBCs) and BMSCs have been shown to contribute to an increase in phosphorylated neurofilament marker SMI-31 and synaptophysin expression. These markers are involved in axonal and synaptic plasticity that promotes white matter remodeling in the ischemic brain (37, 38, 41). Vascular remodeling was revealed by an increase in the expression of smooth muscle actin (α-SMA) and Von Willebrand Factor (vWF) (37) in the ischemic brain which led to an improvement in functional outcomes (37, 38). In addition, BMSCs decreased miR-145 expression, which reduces endothelial cell proliferation, contributing to increased functional cells and restorative effects in type 1 diabetic rats (38). However, contradictory results have been reported by other authors. BMSC treatment by tail vein starting 24h after middle cerebral artery occlusion in diabetes type 1 rats resulted in increased brain hemorrhage, BBB leakage and higher expression of angiogenin. This causes accelerated cerebral arteriosclerosis and prevents improvement in functional outcomes (39).

In subsequent studies, however, the harmful effects of BMSC administration were negated when the treatment was administered in combination with Niaspan. Despite the combination, BMSC and Niaspan treatment for stroke did not improve functional outcomes. However, it did decrease BBB leakage and atherosclerotic-like changes (40) and promoted white matter remodeling in type 1 diabetes rats after stroke (41) (Table 1).

Regarding type 2 diabetes, all experimental animal studies have shown a beneficial effect of MSCs. Independent of treatment with HUCBCs, BMSCs or bone marrow stromal cell-conditioned medium initiated at 24 h or 3 days after stroke via intravenous administration improved functional recovery, promoted restorative effects and reduced BBB disruption after stroke in type 2 diabetes rats (42–46) (Table 1). As in type 1 diabetes, MSC therapy is also associated with white matter remodeling in type 2 diabetes (42, 46), participating in axonal regeneration, sprout and remyelination which led to improved long-term functional outcomes (46). MSC therapy after stroke also contributes to vascular remodeling in type 2 diabetes. Specifically, BMSC-CM treatment enhanced expression of angiopoietin 1 (Ang1), tyrosine-protein kinase receptor Tie-2 (45), α-SMA, and vWF (39, 43), which indicates higher cerebral artery and vascular density (42). Ang1 also seems to be related with a reduction in BBB leakage and promotes vascular stabilization in the ischemic brain. Moreover, it plays a role in white matter remodeling (37, 41), which may improve functional outcome. Regarding the immune system, several authors defend the idea that MSC treatment can also regulate pro-inflammatory factors. This has been shown by a decrease in expression of the receptor for advanced glycation end-products (RAGE) after HUCBC (42) and BMSC (44) treatment in diabetic rats. This indicates a decrease in inflammation, neuronal death, vascular injury and brain damage following ischemia in type 2 diabetic rats. HUCBC and BMSC treatment of type 2 diabetic stroke rats also had an effect on macrophage polarization, promoting decreases inflammation (42, 46), decreased the expression of the proinflammatory protein toll-like receptor 4 (TLR4) (42), increasing brain platelet-derived growth factor (PDGF) expression in the ischemic brain, contributing to restoration (46) and promoting functional improvement after stroke (42).

In summary, with regard to type 1 diabetes, although the experimental studies are homogeneous in terms of the route of administration, cell dose and stroke location, the results are contradictory with no good functional recovery observed in any of them. This reveals the need for further research to increase understanding of the interaction between type 1 diabetes mellitus and cell-based therapy with MSCs. Although all the studies performed thus far reveal that treatment of type 2 diabetes with MSCs in ischemic stroke models can be successful, to our knowledge no studies have been conducted to test the efficacy of MSCs in hemorrhagic stroke. The meager interest aroused could be due to the fact that the prevalence of diabetes is higher in patients with ischemic compared with hemorrhagic stroke, as recently demonstrated by a meta-analysis (61).

### Hyperglycemia in Stroke

Hyperglycemia plays an important role in stroke and is associated with poorer functional recovery and an increase in mortality in ischemic and hemorrhagic stroke (62, 63). It has been observed that after stroke, hyperglycemia is present in over 50% of patients (64). Given the high percentage of patients who develop post-stroke hyperglycemia, it is important to conduct research in hyperglycemic animal models. Two options have been used to reproduce hyperglycemic situations in preclinical stroke models, depending on the researchers' requirements: acute hyperglycemia after anesthesia can be mimicked in animals with intravenous infusion of glucose (65); or intravenous administration of streptozotocin and continuous administration of insulin, in which the animals develop hyperglycemia after 1 week (66). To our knowledge, no data have been published on how MSC-based therapy affects stroke associated with hyperglycemia.

### Obesity in Stroke

Obesity, especially in the abdominal zone (67), is an important risk factor at all ages. An increase in body mass index (BMI) significantly increases the risk of stroke. Compared with healthy weight individuals, the obese population has a 64% greater probability of experiencing an ischemic stroke (68). The association between BMI and ischemic stroke is linear, without differences between sex or race (69). Other studies also linked obesity with hemorrhage, in which people with a high BMI have a 37% higher incidence (70).

Various animal models have been established to induce obesity in rats (71). Yet despite the high prevalence of obesity in stroke, no research has been performed on how MSC therapy affects the pathology of stroke associated with obesity. In conclusion, studies should be conducted to show the interactions between obesity and MSC treatment after stroke.

### Take Home Message

Most of the studies carried out on this subject indeed favor BMMSCs. However, compared to other cell types, adipose tissue-derived mesenchymal stem cells (ADMSCs) have several advantages in clinical applications for neurological disorders (72). ADMSCs are derived from adipose tissue and thus are abundant, accessible and easy to obtain using lipoaspiration techniques. Moreover, they provide proliferation and differentiation potential (73) without adverse side effects (12, 74, 75) and they can be administered without ethical concerns. All of these advantages mean that ADMSCs present a great opportunity for the treatment of diseases such as comorbidities in stroke. Further studies should take this into consideration.

It should be emphasized that patients present not only one but also several associated comorbidities at a time. Therefore, it is clear that there is a need for multimodelling for successful translation of preclinical research to the clinic (6). In this sense, not only modifiable factors, but also non-modifiable risk factors such as age and sex, are important to include in animal model studies (6). Moreover, animal models with co-morbidities show higher variability in outcome measures and therefore, higher sample sizes should be estimated with the specific disease model in mind (6). Also, aged animals take longer to recover after stroke, but eventually recovered to the same degree as young mice, making clear the importance of implementing long-term studies (76).

Adequate selection of the experimental model for stroke and comorbidity induction is important to reduce mortality, as it is often higher in models with preexisting comorbid conditions. This strategy leads to decreased costs. Besides, outcome measures should be optimized and adequate for these studies as there is variability in outcomes compared to healthy animals (6).

## Conclusion

The high prevalence of comorbidities in patients with stroke indicates the need for therapies in preclinical studies that take into account these comorbidities in order to avoid failures in translation to the patient. Preclinical studies are beginning to evaluate the efficacy of MSC treatment in stroke associated with comorbidities, especially hypertension, for ischemic and hemorrhagic stroke. Regarding aging and diabetes, only ischemic stroke studies have been performed. For the moment, few studies have been performed and contradictory results are being reported. These contradictory results may be due to the use of different stroke and comorbidity models, and to the use of different protocols for administering cell-based therapies. This situation indicates a further need to promote standardization of cell concentration and administration routes. Obesity and hyperglycemia have been completely ignored, although they are frequently present in patients with stroke. For this reason, the role of comorbidities should have a more prominent place in preclinical stroke studies. This will hopefully improve bench-to-bedside translation and identify viable therapeutic options.

## Author Contributions

FL-G and LD wrote the first draft of the manuscript. MG-dF and LO-O wrote sections of the manuscript. BF, GR-A, and ED-T contributed to manuscript revision and read and approved the submitted version. MG-F contributed to the conception of the study, wrote and revised the manuscript, and approved the submitted version.

### Conflict of Interest Statement

The authors declare that the research was conducted in the absence of any commercial or financial relationships that could be construed as a potential conflict of interest.

## Abbreviations

ICH: intracerebral hemorrhage
MSCs: mesenchymal stem cells
BBB: blood-brain barrier
BMMNCs: bone marrow mononuclear cells
BMSCs: bone marrow stromal cells
G-CSF: granulocyte colony-stimulating factor
BMMSCs: bone marrow mesenchymal stem cells
SHRs: spontaneously hypertensive rats
SHR-SPs: stroke-prone SHRs
HUCBCs: human umbilical cord blood cells
DM-BMSCs: BMSCs derived from type I diabetes rats.
